# Footprint-based functional analysis of multiomic data

**DOI:** 10.1016/j.coisb.2019.04.002

**Published:** 2019-06

**Authors:** Aurelien Dugourd, Julio Saez-Rodriguez

**Affiliations:** 1 Heidelberg University, Faculty of Medicine, and Heidelberg University Hospital, Institute of Computational Biomedicine, Bioquant, 69120 Heidelberg, Germany; 2 RWTH Aachen University, Faculty of Medicine, Joint Research Centre for Computational Biomedicine (JRC-COMBINE), 52074, Aachen, Germany

**Keywords:** Transcriptomics, Phosphoproteomics, Proteomics, Metabolomics, Multi-omics, Trans-omics, Footprint, Mechanistic, Data analysis, Functional, Integration

## Abstract

Omic technologies allow us to generate extensive data, including transcriptomic, proteomic, phosphoproteomic and metabolomic. These data can be used to study signal transduction, gene regulation and metabolism. In this review, we summarise resources and methods to analysis these types of data. We focus on methods developed to recover functional insights using footprints. Footprints are signatures defined by the effect of molecules or processes of interest. They integrate information from multiple measurements whose abundances are under the influence of a common regulator. For example, transcripts controlled by a transcription factor or peptides phosphorylated by a kinase. Footprints can also be generalised across multiple types of omic data. Thus, we also present methods to integrate multiple types of omic data and features (such as the ones derived from footprints) together. We highlight some examples of studies that leverage such approaches to discover new biological mechanisms.

## Highlights

• Functional information on signalling pathways, metabolism and gene regulation can be found across multiple types of omic data.
• One way to extract such information is to consider these data as the footprint of the activity of enzymes and pathways.
• Information on enzyme/pathway activities and omic data can be integrated together to contextualise multi-scale networks.
• Such an approach can lead to the discovery of regulatory events spanning across multiple biological processes.

## Graphical abstract

**Figure:**
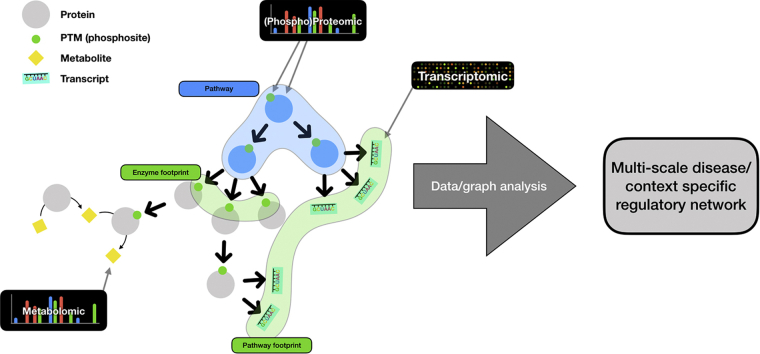

## Introduction

**Figure 1:**
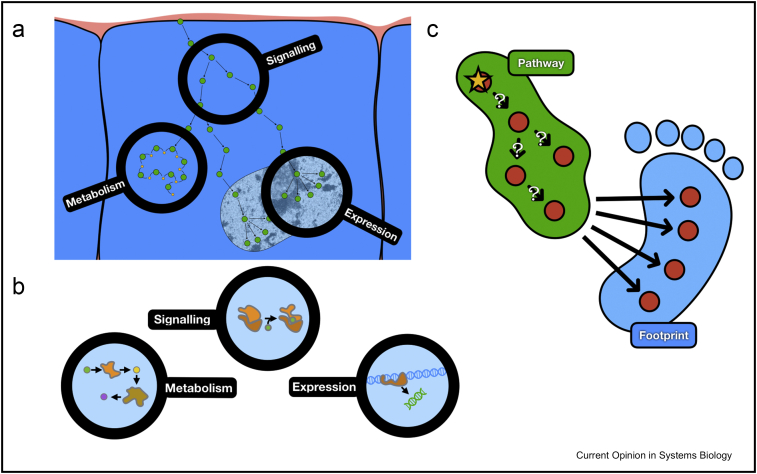
From pathway to footprint for functional analysis of omic data. **(a, b)** Schematic representation of the interactions between signalling, gene regulation and metabolism. The main types of omic data to study are highlighted. **(c)** A certain pathway (green) and the potential footprint of perturbing this pathway (blue). The question marks represent the uncertainty of the functionality of interaction in the pathway in a specific context.

In a cell, numerous molecules are constantly interacting and reacting to adapt to the environment and preserve homoeostasis. These molecules can be separated in distinct classes, mostly DNA, RNA of various natures (messenger RNA, microRNA, etc), proteins and metabolites. They can be subjected to various chemical modifications such as methylation, phosphorylation, ubiquitinilation or glycosylation. Each of these modifications can affect the physical properties of these molecules and, consequently, their functions. In particular, modifications of proteins are often organised in cascades. These cascades are interlinked, forming a complex network that controls most cellular functions. Over the past decades, subparts of this network have been characterised and defined according to the types of reactions and molecules interacting together, notably signalling pathways, regulatory networks and metabolic networks. Roughly, signalling and regulatory networks represent subnetworks composed mainly of kinases, phosphatases and transcription factors (TFs) connecting proteic sensors (such as membrane receptors) to gene expression. Kinases are responsible for the phosphorylation of proteins, whereas TFs, which are also interconnected, regulate the abundance of RNA transcripts. Metabolic networks are mainly composed of small molecules (metabolites) that are transformed into one another through reactions catalysed by metabolic enzymes (Figure 1 a and b). Thus, changes in the abundance of phosphorylated proteins, transcripts and metabolites hold information about the functional states of signalling, regulatory and metabolic networks, respectively.

Today, it is possible to measure the abundance of thousands of RNA transcripts, protein peptides (chemically modified or not) and metabolites. Such data sets, along with the systematic characterisation of other biomolecules (e.g. lipidomics, genomics), are referred to as omic data sets. All these abundances can be considered as the molecular signature of a biological sample in a specific condition, for example, cells treated with an enzymatic inhibitor. This concept can also be scaled down at the level of specific enzymes, such as TFs or kinases; the abundances of the target transcripts of a TF can be viewed as the footprint of the TF activity. The same concept applies to the target phosphopeptides of a kinase. A footprint can also be derived for a pathway or process and inform us on their activity. In a classic ‘mapping’ strategy, the activity of a pathway is inferred from measurements of its own components and the activity of enzymes is estimated from measurements of their corresponding transcripts/proteins. In contrast, footprint-based strategies estimate activities from molecular readouts considered to be downstream of the pathway/enzyme (Figure 1 c).

In this review, we will cover recent methods to analyse and extract relevant functional and mechanistic information using molecular signatures applied to omic data. We will also present strategies to integrate together multiple types of omic data. We will focus mainly on molecular measurements directly related to signalling pathways and metabolic reaction networks that can be obtained from transcriptomic, (phospho)proteomic and metabolomic data. We leave other omic data out of the scope of the review, in particular, (epi)genomic. Accordingly, we will describe features derived from this data, in particular, using footprints. First, we present different types of online knowledge databases that can be used to extract functional insights from omic data sets. Then, we summarise mapping and footprint methods, as well as network-based approaches. Finally, we will discuss how these methods can be used to integrate together different types of omic data sets.

## Prior knowledge resources

### Ontologies and protein–protein interaction databases

A powerful strategy for the analysis of any omic data set is to integrate it with the current knowledge of the underlying biology. This knowledge is available in multiple resources [1], see Table 1. For example, gene ontology [2] is arguably the most used resource for gene annotation. These annotations are very useful to quickly get an overview of molecular functions, cellular compartments and biological processes associated with specific genes. Many other types of annotations, such as signalling pathways, cancer hallmarks, chemical and genetic perturbation signatures, are available in databases such as MSigDB [3]. Large resources for protein–protein interactions (PPIs) are also available [1]. For example, STRINGdb [4] pulls together many different sources of PPIs, from experimentally validated interactions to automatic literature search, whereas Omnipath [5] focuses on databases of curated interactions.

**Table 1:** Selected prior knowledge resources discussed in this review.

Database	Content	Link
Brenda	Metabolic enzyme–substrate interactions, reaction networks and enzyme structures.	https://www.brenda-enzymes.org/
CophosK	Kinase–substrate interaction inference.	http://compbio.case.edu/omics/software/cophosk/
Gene Ontology	Molecular functions, biological processes and cellular components	http://geneontology.org/
KEA2	Kinase–substrate interactions from multiple resources.	http://www.maayanlab.net/KEA2/index.html
KEGG	Metabolic enzyme–substrate interactions and reaction networks.	https://www.genome.jp/kegg/
Kinomexplorer	Kinase–substrate interaction inference.	http://kinomexplorer.info/
MSigDB	Gene sets of hallmarks, positions, pathways and perturbation signatures, motifs, gene ontology, oncogenic and immunologic.	http://software.broadinstitute.org/gsea/msigdb
Omnipath	Protein–protein interactions pulled from various resources (mainly curated). Kinase/substrate interactions. Transcription factor–target interactions (DoRothEA).	http://omnipathdb.org/
Pathway commons	Signalling and metabolic pathways from various databases.	http://www.pathwaycommons.org/
PTMSigDB	Posttranslational modification signatures.	https://github.com/broadinstitute/ssGSEA2.0
Reactome	Metabolic enzyme–substrate interactions and reaction networks.	https://reactome.org/
STITCHdb	Chemical–proteins interactions.	http://stitch.embl.de/
STRIBGdb	Protein–protein interactions pulled from various resources (curated and inferred).	https://string-db.org/
Transfac	Transcription factor–target interactions. (Commercial)	http://gene-regulation.com/pub/databases.html
TRRUST	Transcription factor–target interaction.	https://www.grnpedia.org/trrust/

### Enzyme/substrate databases

Databases that capture relationships between enzymes and their substrates are useful to extract relevant information about enzymes from transcriptomic and phosphoproteomic data. These relationships are either predicted with computational methods or experimentally validated.

TF targets are available in databases such as TRANSFAC [6] or TRRUST [7]. TRRUST uses consensus sequence pattern search to infer potential TF targets, and some of these interactions may be experimentally validated. Hence, the level of confidence in a TF-target interaction can vary. DoRothEA [8], which is also embedded in Omnipa [5], integrates multiple TF target resources (including TRRUST). DoRothEA annotates TF-target interactions with a confidence index based on the source of the interaction (pattern search, experimental validation, etc). Higher confidence interactions such as experimentally validated ones seem to yield better estimations of TF activity [8].

Similar databases exist for kinases. PhosphositePlus [9] contains curated information about phosphosites such as their function and kinase/substrate interactions. PTMSigDB [10], a database of posttranslational modification signatures, combines consensual perturbation footprint across thousands of phosphoproteomic data sets, curated kinase targets and pathways. KinomeExplorer [11] infers substrate of kinases with amino acid pattern search and known PPIs. CophosK [12] complements experimentally validated databases with correlated phosphosite based on phosphoproteomic data, thus creating context specific kinase/substrate networks. KEA2 [13] and Omnipath [5] combine together multiple databases of kinase/substrate interactions.

Finally, information on metabolic enzymes and their targeted metabolites exists in resources such as KEGG [14], Brenda [15], Reactome [16] and REcon3D [17].

### Multilevel interaction databases

Some multilevel interaction databases (spanning across multiple different biological process) already exist. STITCH [18], a complement of STRING, combines together interactions between chemicals and proteins with PPIs. Omnipath combines TF/targets, kinase/substrate, PPIs and drugs. Pathway Commons combines signalling and metabolic pathways from various databases [19]. In the future, it is likely that more databases that combine together multiple types of molecular interactions will appear. As more multiomic data sets are generated, the importance of such combinations of resources will increase.

## Gene set and pathway enrichment analysis

**Figure 2:**
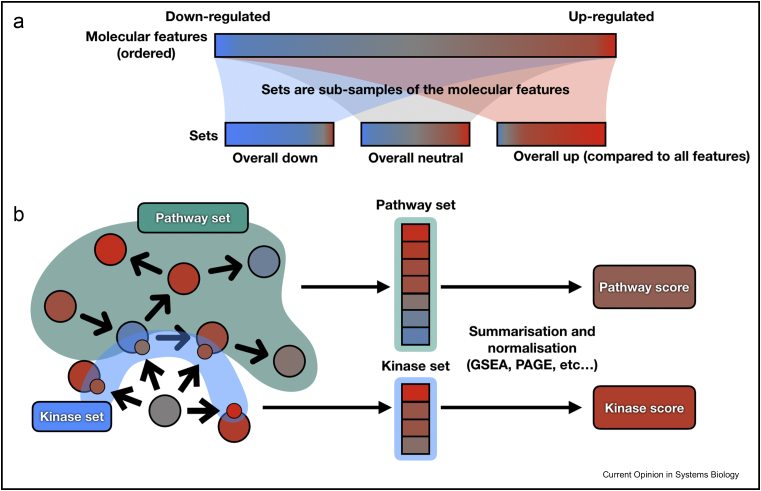
Comparison between pathway and kinase enrichment analysis. **(a)** Simplified representation of the fundamental idea of statistical enrichment analysis. Pathways, gene annotation and enzyme targets are sets of molecular features. The goal of an enrichment analysis is to characterise the significance of an overall change of each set compared to the rest of all measured molecular features in a specific condition. **(b)** In a classic pathway enrichment analysis, the features used to compute the enrichment scores are the members of the pathway itself. In contrast, a kinase enrichment analysis computes the enrichment score with targets of the kinase, but not the kinase itself. The same principle applies for transcription factor and pathway footprint enrichment analysis. GSEA, gene set enrichment analysis; PAGE, parametric analysis of gene set enrichment.

Gene sets are groups of genes that share a common characteristic (for example, genes that participate in the same biological process). These are available in annotation resources described in 2. Prior knowledge resources. Gene sets can be analysed using multiple methods that can be largely classified as either overrepresentation or enrichment analysis. Overrepresentation analysis (ORA) usually tries to answer the following question: when comparing genes differentially expressed between two conditions, are there sets of genes that contain significantly more differentially expressed genes than expected? Statistical enrichment analysis (EA; often referred to as gene set enrichment analysis (GSEA)-like approaches) tries to answer a slightly different question: when comparing genes differentially expressed between two conditions, are there some sets in which the overall difference of expression is more extreme than expected? EA approaches do so by summarising measurement-level statistics (e.g. fold-changes, t-values, p-values) belonging to the same group/set into a single score and estimate if this summarised score is significantly more extreme than expected (Figure 2 a, see 4. Footprint analysis for a concrete example and [20]). While they answer slightly different questions, EA has the advantage that it does not require to decide a-priori which genes are significantly changed or not. DAVID [21] is widely used to run gene set analysis using ORA with gene ontology. GSEA [22] and parametric analysis of gene set enrichment [23] are examples of statistical enrichment analysis tools. EnrichR [24] is a popular platform that provides an intuitive user interface to perform gene set analysis with ORA or EA methods. These tools can also be used with pathway ontologies such as the one present in MSigDB [3] to perform pathway enrichment analysis (Figure 2b). Recent developments in EA take advantage of the underlying topology of pathway. This is done either in a data-driven manner based on correlation between measurements of the same set [25] or using prior knowledge of interactions between members of a pathway [26].

Originally, gene set/pathway enrichment analysis was mainly used to assess whether a specific gene annotation is significantly enriched with extremely deregulated genes. However, this method is very flexible and can be adapted for many different uses. For example, associations between drugs and their expression signature (such as those found in LINCS L1000 [27] and DSigDB [28]) can be used to identify and repurpose drugs with transcriptome and/or proteomic data.

## Footprint analysis

**Figure 3:**
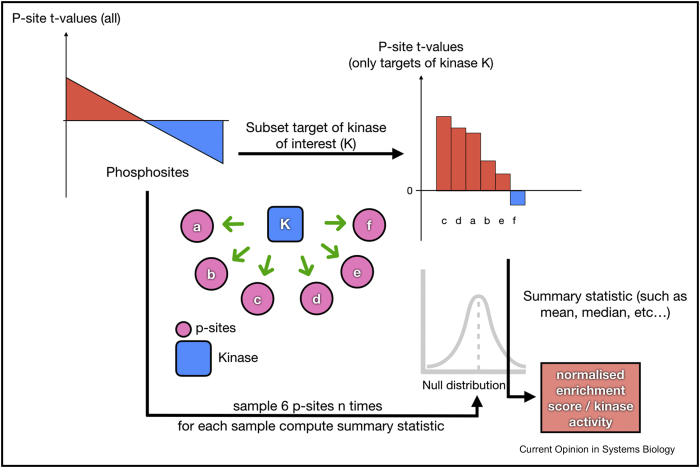
Example of kinase activity estimation with statistical enrichment analysis. Consider an experiment where the changes in phosphosite abundance were measured between two specific conditions. Given a kinase K that can phosphorylate six phosphosites **(a, b, c, d, e, f)**, one could assume that the changes in abundance of the six phosphosites mirror changes in the activity of kinase K. To estimate this change of activity, the statistics (t-values in this example) associated with the change of abundance of the six targets of kinase K are summarised (using e.g. mean or variance). This summary statistic is called the enrichment score. Then, we need to estimate whether this enrichment score is significantly different from what would be expected from any given set of six phosphosites. To this end, six phosphosites are sampled randomly n times from all the phosphosites available in this study to generate a null distribution of enrichment scores. The enrichment score of kinase K is then normalised with this distribution. Thus, the resulting normalised enrichment score represents how extreme the change in the activity of kinase K is compared with possible kinases randomly associated to phosphosites.

EA approaches can also be used for footprint analysis, such as TF and kinase enrichment analysis. Even though the algorithm is the same as for pathway enrichment analysis, the prior knowledge sources are sets of enzyme targets, fundamentally changing the interpretation and usefulness of enrichment scores. This is possible because, in the case of EA approaches, the enrichment score of a given set directly summarises the changes of the members of the set. Thus, an enrichment score obtained from a set of functional targets of an enzyme can be interpreted directly as a proxy of the activity of this enzyme (Figure 2b). An example of the procedure to estimate the activity of a kinase with statistical enrichment is shown in Figure 3.

### TF activity

VIPER is an enrichment analysis method building up on parametric analysis of gene set enrichment. VIPER can estimate the activity of proteins, typically TFs, using the abundance changes of their targets as a proxy of their activity [29]. Originally, VIPER uses data-driven inferred TF-targets interactions, but any type of set collection can be used, and it has been applied to the aforementioned DoRothEA TF-targets interactions [30].

Osmanbeyoglu et al. [31] developed an approach based on bilinear regression to estimate the activity of TFs with transcriptomic and phosphoproteomic. This approach directly accounts for phosphorylation events measured upstream of TFs when estimating their activity change.

### Kinase activity

Analogously to TFs, the activity of kinases can be estimated from the abundance changes of their substrates from phosphoproteomic data. As for TFs, different statistical models can be used, for example KSEA 32, •33 or KinasePA [34], an approach specifically tailored to handle data sets with more than two conditions. In Ref. [35], kinase activity change estimations obtained from various statistical models were compared with kinases knockout and ligand perturbation data sets. It was shown in this context that simple statistics of the footprint can displayed slightly better agreement with experimental data than more complex statistics such as GSEA or multivariate linear regression models. Yet, the quality of the target set collection seemed to be the main determinant of performance.

### Pathway activity

Tools presented in 3. Gene set and pathway enrichment analysis can yield insight about the activity of pathways using gene expression data [36]. However, these approaches remain limited by the fact that the expression of a gene only partially correlates with the activity of the corresponding protein in a pathway [37]. This limits the amount of information that can be retrieved about the functional state of a pathway from expression measurements related to the members of the pathway itself. An alternative approach is to estimate the activity of the pathway by looking at the genes that are known to change when the pathway is activated or inhibited, akin the footprint methods for kinases and TFs. PROGENy [38] (an extension of SPEED://paperpile.com/c/vfuz6S/Lih3F [39]) learns transcriptomic footprints of a specific pathway from multiple experiments where the pathway is perturbed. Such footprints represent indirect targets downstream of the pathway. They can then be used with the same algorithms presented in 4.1. TF activity and 4.2 Kinase activity. These footprint gene sets have been shown to be more informative than the mapping/ontology gene sets 38, ••40.

## Multiscale networks

### Correlation-based methods for multiomic integration

Joint analysis of omic data sets allow us to study the interactions between biological processes. The arguably simplest and most intuitive approach is to use correlation-based methods: correlation between different omic measurements across samples suggests that the processes reflected in one omic regulate the processes reflected by the other or that there is coregulation by a third (often unknown) process. This makes it possible to reconstruct networks of interactions based on correlations between multiple measurements and features. For example, correlations between metabolite and metabolic enzyme transcript abundance were estimated in Ref. [41]. This enabled to find mRNA predictors of metabolic abundances. The predicted abundances of these metabolites were, in turn, good predictors of cancer patient survival. A combination of principal component analysis and partial correlation was also used to systematically find pairs of metabolites that are coregulated by either transcriptional or posttranscriptional mechanisms [42]. Multi-Omic Factor Analysis (MOFA) is a method that generalise principal component analysis to handle multiple omic data [43]. The method was originally applied on a data set including somatic mutations, RNA expression and DNA methylation but is, in principle, applicable to other type of omic data sets such as proteomic, phosphoproteomic and metabolomic and their corresponding footprints (e.g. kinase and TF activities).

Indeed, correlation based approaches can also be used downstream of footprint analysis to connect activity scores with other measurements. For example, kinase activities estimated from phosphoproteomics were correlated with metabolites to find kinases that regulate the activity of metabolic enzymes through posttranslational modifications [44].

### Network contextualisation

Most network resources (such as the ones presented in 2. Prior knowledge resources) are generic. They recapitulate all known interactions between omic data in different organisms. However, not all proteins are expressed in all types of cell. Different mutational backgrounds, especially in cancer, can also alter the properties of proteins, such as enzymatic activity and binding ability. Thus, various tools exist to contextualise networks according to specific conditions 45, 46. These methods combine protein interactions and omic data sets to find significantly deregulated subsets of a larger interaction network. They usually rely on a static protein–protein interaction network and graph theory. Alternative approaches find the most coherent subnetwork connecting perturbation targets (i.e. known proteins that are altered in some way) with deregulated transcripts https://www.biorxiv.org/content/10.1101/541888v1 47, •48. To do so, protein networks are abstracted as causal models, where nodes (proteins) and edges (interactions) can be active or not. Then, the signed subnetworks that lead to the best fit between its output and experimental measurements are identified. A similar approach was also used in the context of phosphoproteomic data to reconstruct signalling pathways from a generic kinase/substrate network 49, 50. The pathways reconstructed in this way often share similarities with canonical pathways. However, because they use generic prior knowledge networks, they can include nodes that are usually absent from canonical pathways.

In the future, it is likely that such approaches will be generalised to directly integrate multiple type of omic measurements at the same time, combining both measurements and/or output of footprint analysis. In fact, there are already a few examples of recent methods to contextualise networks with multiple types of omic data. The prize-collecting Steiner forest algorithm has been used to find optimal subnetworks in a prior combination of PPI and reaction network based on metabolic and protein abundance measurements [51]. The TieDIE [52] algorithm can contextualise signalling pathway with specific type of cancer based on transcriptomic and phosphoproteomic data. A pipeline developed by Huna et al. [53] first extracts relevant metabolic pathways based on metabolomic data and then overlay proteomic and transcriptomic data on these subnetworks. Finally, the HotNet [54] algorithm generalises approaches based on graph theory [45] to find altered subnetwork across multiple biological scales and integrate different types of omic data together.

## Multiomic network to find potential actionable treatment targets

**Figure 4:**
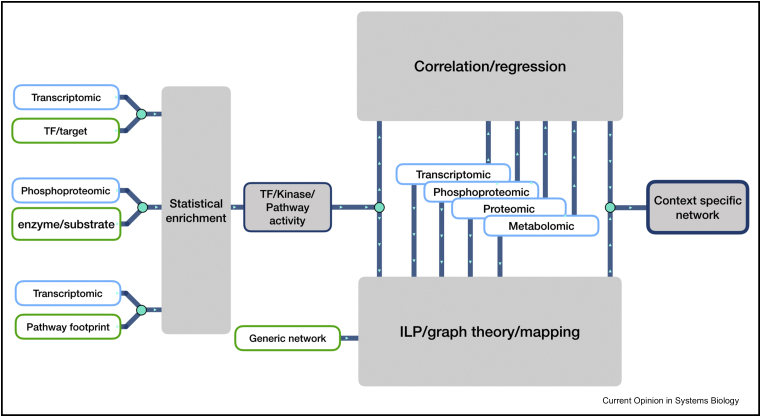
Summarised representation of the multiomic analysis workflow. On the left, statistical enrichment analysis is used to estimate activity of kinases, transcription factors and pathways. Then, multiple types of omic data can be connected together with these activities by correlation/regression methods. They can also be combined with prior knowledge network through network contextualisation methods (optimisation, graph theory and mapping). Finally, the output of network contextualisation and correlation-based methods can be used, independently or combined, to generate multiomic context-specific networks.

To conclude, we believe that integrating multiple types of omic data together using biological knowledge and appropriate computational models will allow us to better understand cellular mechanisms in many contexts (Figure 4). Novel type of regulatory mechanism in *Escherichia coli* has been discovered by integrating genomic, transcriptomic, ribosomal profiling, proteomic and metabolomic data [55]. Global network reprogramming events occurring in diabetes have been studied by simultaneously looking at transcriptomic, proteomic, phosphoproteomic and metabolomic changes over a time course [56]. Posttranslation regulatory mechanism in fumarate hydratase deficient cancer cells were decoded by integrating proteomic, phosphoproteomic and metabolomic data together [57]. These three studies illustrate how generating multiple parallel omic data sets targeted toward signalling pathway and metabolism can yield very valuable insight to understand the molecular features of diseases. In the future, it is very likely that more multi-omic data sets will be generated to reconstruct a global regulatory picture of cellular functions. The methods discussed in 4. Footprint analysis and 5. Multiscale networks can be useful for the analysis of such multi-omic data sets. They can generate insights into cellular mechanisms spanning across signalling, regulatory and metabolic networks. Indeed, these methods mainly rely on principles that are conserved across signalling and metabolism, such as enzyme/substrate relationships, and are specifically designed to provide functional insights.

## Author contributions

Aurelien Dugourd wrote the manuscript under the supervision of Julio Saez-Rodriguez.

## Conflict of interest

Nothing declared.

## References

[1] Miryala S.K., Anbarasu A., Ramaiah S. (2018). Discerning molecular interactions: a comprehensive review on biomolecular interaction databases and network analysis tools. Gene.

[2] Ashburner M., Ball C.A., Blake J.A., Botstein D., Butler H., Cherry J.M., Davis A.P., Dolinski K., Dwight S.S., Eppig J.T. (2000). Gene ontology: tool for the unification of biology. The Gene Ontology Consortium. Nat Genet.

[•3] Liberzon A., Subramanian A., Pinchback R., Thorvaldsdóttir H., Tamayo P., Mesirov J.P. (2011). Molecular signatures database (MSigDB) 3.0. Bioinformatics.

[4] Szklarczyk D., Franceschini A., Wyder S., Forslund K., Heller D., Huerta-Cepas J., Simonovic M., Roth A., Santos A., Tsafou K.P. (2015). STRING v10: protein–protein interaction networks, integrated over the tree of life. Nucleic Acids Res.

[5] Türei D., Korcsmáros T., Saez-Rodriguez J. (2016). OmniPath: guidelines and gateway for literature-curated signaling pathway resources. Nat Methods.

[6] Matys V., Kel-Margoulis O.V., Fricke E., Liebich I., Land S., Barre-Dirrie A., Reuter I., Chekmenev D., Krull M., Hornischer K. (2006). TRANSFAC® and its module TRANSCompel®: transcriptional gene regulation in eukaryotes. Nucleic Acids Res.

[7] Han H., Cho J.-W., Lee S., Yun A., Kim H., Bae D., Yang S., Kim C.Y., Lee M., Kim E. (2018). TRRUST v2: an expanded reference database of human and mouse transcriptional regulatory interactions. Nucleic Acids Res.

[8] Garcia-Alonso L., Ibrahim M.M., Turei D., Saez-Rodriguez J. (2018). Benchmark and integration of resources for the estimation of human transcription factor activities. bioRxiv.

[9] Hornbeck P.V., Kornhauser J.M., Tkachev S., Zhang B., Skrzypek E., Murray B., Latham V., Sullivan M. (2012). PhosphoSitePlus: a comprehensive resource for investigating the structure and function of experimentally determined post-translational modifications in man and mouse. Nucleic Acids Res.

[••10] Krug K., Mertins P., Zhang B., Hornbeck P., Raju R., Ahmad R., Szucs M., Mundt F., Forestier D., Jane-Valbuena J. (2018). A curated resource for phosphosite-specific signature analysis. Mol Cell Proteomics.

[11] Horn H., Schoof E.M., Kim J., Robin X., Miller M.L., Diella F., Palma A., Cesareni G., Jensen L.J., Linding R. (2014). KinomeXplorer: an integrated platform for kinome biology studies. Nat Methods.

[12] Ayati M., Wiredja D., Schlatzer D., Maxwell S., Li M., Koyuturk M., Chance M., CoPhosK (2018). A method for comprehensive kinase substrate annotation using Co-phosphorylation analysis. bioRxiv.

[13] Lachmann A., Ma’ayan A. (2009). KEA: kinase enrichment analysis. Bioinformatics.

[14] Kanehisa M., Goto S. (2000). KEGG: kyoto encyclopedia of genes and genomes. Nucleic Acids Res.

[15] Jeske L., Placzek S., Schomburg I., Chang A., Schomburg D. (2019). BRENDA in 2019: a European ELIXIR core data resource. Nucleic Acids Res.

[16] Fabregat A., Jupe S., Matthews L., Sidiropoulos K., Gillespie M., Garapati P., Haw R., Jassal B., Korninger F., May B. (2018). The reactome pathway knowledgebase. Nucleic Acids Res.

[17] Brunk E., Sahoo S., Zielinski D.C., Altunkaya A., Dräger A., Mih N., Gatto F., Nilsson A., Preciat Gonzalez G.A., Aurich M.K. (2018). Recon3D enables a three-dimensional view of gene variation in human metabolism. Nat Biotechnol.

[18] Szklarczyk D., Santos A., von Mering C., Jensen L.J., Bork P., Kuhn M. (2016). STITCH 5: augmenting protein–chemical interaction networks with tissue and affinity data. Nucleic Acids Res.

[19] Cerami E.G., Gross B.E., Demir E., Rodchenkov I., Babur O., Anwar N., Schultz N., Bader G.D., Sander C. (2011). Pathway Commons, a web resource for biological pathway data. Nucleic Acids Res.

[••20] Ackermann M., Strimmer K. (2009). A general modular framework for gene set enrichment analysis. BMC Bioinf.

[21] Huang D.W., Sherman B.T., Lempicki R.A. (2008). Systematic and integrative analysis of large gene lists using DAVID bioinformatics resources. Nat Protoc.

[22] Subramanian A., Tamayo P., Mootha V.K., Mukherjee S., Ebert B.L., Gillette M.A., Paulovich A., Pomeroy S.L., Golub T.R., Lander E.S. (2005). Gene set enrichment analysis: a knowledge-based approach for interpreting genome-wide expression profiles. Proc Natl Acad Sci U S A.

[23] Kim S.-Y., Volsky D.J. (2005). PAGE: parametric analysis of gene set enrichment. BMC Bioinf.

[24] Kuleshov M.V., Jones M.R., Rouillard A.D., Fernandez N.F., Duan Q., Wang Z., Koplev S., Jenkins S.L., Jagodnik K.M., Lachmann A. (2016). Enrichr: a comprehensive gene set enrichment analysis web server 2016 update. Nucleic Acids Res.

[25] Alhamdoosh M., Ng M., Wilson N.J., Sheridan J.M., Huynh H., Wilson M.J., Ritchie M.E. (2017). Combining multiple tools outperforms individual methods in gene set enrichment analyses. Bioinformatics.

[26] Amadoz A., Hidalgo M.R., Çubuk C., Carbonell-Caballero J., Dopazo J. (2018). A comparison of mechanistic signaling pathway activity analysis methods. Briefings Bioinf.

[27] Subramanian A., Narayan R., Corsello S.M., Peck D.D., Natoli T.E., Lu X., Gould J., Davis J.F., Tubelli A.A., Asiedu J.K. (2017). A next generation connectivity map: L1000 platform and the first 1,000,000 profiles. Cell.

[28] Yoo M., Shin J., Kim J., Ryall K.A., Lee K., Lee S., Jeon M., Kang J., Tan A.C. (2015). DSigDB: drug signatures database for gene set analysis. Bioinformatics.

[•29] Alvarez M.J., Shen Y., Giorgi F.M., Lachmann A., Ding B.B., Ye B.H., Califano A. (2016). Functional characterization of somatic mutations in cancer using network-based inference of protein activity. Nat Genet.

[30] Garcia-Alonso L., Iorio F., Matchan A., Fonseca N., Jaaks P., Peat G., Pignatelli M., Falcone F., Benes C.H., Dunham I. (2018). Transcription factor Activities enhance markers of drug sensitivity in cancer. Cancer Res.

[31] Osmanbeyoglu H.U., Toska E., Chan C., Baselga J., Leslie C.S. (2017). Pancancer modelling predicts the context-specific impact of somatic mutations on transcriptional programs. Nat Commun.

[32] Wiredja D.D., Koyutürk M., Chance M.R. (2017). The KSEA App: a web-based tool for kinase activity inference from quantitative phosphoproteomics. Bioinformatics.

[•33] Casado P., Rodriguez-Prados J.-C., Cosulich S.C., Guichard S., Vanhaesebroeck B., Joel S., Cutillas P.R. (2013). Kinase-substrate enrichment analysis provides insights into the heterogeneity of signaling pathway activation in leukemia cells. Sci Signal.

[34] Yang P., Patrick E., Humphrey S.J., Ghazanfar S., James D.E., Jothi R., Yang J.Y.H., KinasePA (2016). Phosphoproteomics data annotation using hypothesis driven kinase perturbation analysis. Proteomics.

[35] Hernandez-Armenta C., Ochoa D., Gonçalves E., Saez-Rodriguez J., Beltrao P. (2017). Benchmarking substrate-based kinase activity inference using phosphoproteomic data. Bioinformatics.

[36] Lim S., Lee S., Jung I., Rhee S., Kim S. (2018). Comprehensive and critical evaluation of individualized pathway activity measurement tools on pan-cancer data. Briefings Bioinf.

[37] Krawczenko A., Bielawska-Pohl A., Wojtowicz K., Jura R., Paprocka M., Wojdat E., Kozłowska U., Klimczak A., Grillon C., Kieda C. (2017). Expression and activity of multidrug resistance proteins in mature endothelial cells and their precursors: a challenging correlation. PLoS One.

[38] Schubert M., Klinger B., Klünemann M., Sieber A., Uhlitz F., Sauer S., Garnett M.J., Blüthgen N., Saez-Rodriguez J. (2018). Perturbation-response genes reveal signaling footprints in cancer gene expression. Nat Commun.

[39] Parikh J.R., Klinger B., Xia Y., Marto J.A., Blüthgen N. (2010). Discovering causal signaling pathways through gene-expression patterns. Nucleic Acids Res.

[••40] Cantini L., Calzone L., Martignetti L., Rydenfelt M., Blüthgen N., Barillot E., Zinovyev A. (2018). Classification of gene signatures for their information value and functional redundancy. NPJ Syst Biol Appl.

[41] Auslander N., Yizhak K., Weinstock A., Budhu A., Tang W., Wang X.W., Ambs S., Ruppin E. (2016). A joint analysis of transcriptomic and metabolomic data uncovers enhanced enzyme-metabolite coupling in breast cancer. Sci Rep.

[42] Schwahn K., Nikoloski Z. (2018). Data reduction approaches for dissecting transcriptional effects on metabolism. Front Plant Sci.

[••43] Argelaguet R., Velten B., Arnol D., Dietrich S., Zenz T., Marioni J.C., Buettner F., Huber W., Stegle O. (2018). Multi-Omics Factor Analysis-a framework for unsupervised integration of multi-omics data sets. Mol Syst Biol.

[•44] Gonçalves E., Raguz Nakic Z., Zampieri M., Wagih O., Ochoa D., Sauer U., Beltrao P., Saez-Rodriguez J. (2017). Systematic analysis of transcriptional and post-transcriptional regulation of metabolism in yeast. PLoS Comput Biol.

[45] Chen B., Fan W., Liu J., Wu F.-X. (2014). Identifying protein complexes and functional modules--from static PPI networks to dynamic PPI networks. Briefings Bioinf.

[46] Á Tényi, de Atauri P., Gomez-Cabrero D., Cano I., Clarke K., Falciani F., Cascante M., Roca J., Maier D. (2016). ChainRank, a chain prioritisation method for contextualisation of biological networks. BMC Bioinf.

[47] Liu A., Trairatphisan P., Gjerga E., Didangelos A., Barratt J., Saez-Rodriguez J. (2019). From expression footprints to causal pathways: contextualizing large signaling networks with CARNIVAL. bioRxiv.

[•48] Bradley G., Barrett S.J. (2017). CausalR: extracting mechanistic sense from genome scale data. Bioinformatics.

[49] Terfve C.D.A., Wilkes E.H., Casado P., Cutillas P.R., Saez-Rodriguez J. (2015). Large-scale models of signal propagation in human cells derived from discovery phosphoproteomic data. Nat Commun.

[50] Köksal A.S., Beck K., Cronin D.R., McKenna A., Camp N.D., Srivastava S., MacGilvray M.E., Bodík R., Wolf-Yadlin A., Fraenkel E. (2018). Synthesizing signaling pathways from temporal phosphoproteomic data. Cell Rep.

[51] Pirhaji L., Milani P., Leidl M., Curran T., Avila-Pacheco J., Clish C.B., White F.M., Saghatelian A., Fraenkel E. (2016). Revealing disease-associated pathways by network integration of untargeted metabolomics. Nat Methods.

[52] Drake J.M., Paull E.O., Graham N.A., Lee J.K., Smith B.A., Titz B., Stoyanova T., Faltermeier C.M., Uzunangelov V., Carlin D.E. (2016). Phosphoproteome integration reveals patient-specific networks in prostate cancer. Cell.

[53] Huan T., Palermo A., Ivanisevic J., Rinehart D., Edler D., Phommavongsay T., Benton H.P., Guijas C., Domingo-Almenara X., Warth B. (2018). Autonomous multimodal metabolomics data integration for comprehensive pathway analysis and systems biology. Anal Chem.

[54] Reyna M.A., Leiserson M.D.M., Raphael B.J. (2018). Hierarchical HotNet: identifying hierarchies of altered subnetworks. Bioinformatics.

[55] Ebrahim A., Brunk E., Tan J., O'Brien E.J., Kim D., Szubin R., Lerman J.A., Lechner A., Sastry A., Bordbar A. (2016). Multi-omic data integration enables discovery of hidden biological regularities. Nat Commun.

[•56] Kawata K., Hatano A., Yugi K., Kubota H., Sano T., Fujii M., Tomizawa Y., Kokaji T., Tanaka K.Y., Uda S. (2018). Trans-omic analysis reveals selective responses to induced and basal insulin across signaling, transcriptional, and metabolic networks. iScience.

[57] Gonçalves E., Sciacovelli M., Costa A.S.H., Tran M.G.B., Johnson T.I., Machado D., Frezza C., Saez-Rodriguez J. (2018). Post-translational regulation of metabolism in fumarate hydratase deficient cancer cells. Metab Eng.

